# Hypersalivation and coarse tremors as uncommon side effects of acamprosate: A case report

**DOI:** 10.1192/j.eurpsy.2021.2059

**Published:** 2021-08-13

**Authors:** D. Bhandutia, S. Nayok

**Affiliations:** Psychiatry, Sri Siddhartha Medical College, Tumakuru, India

**Keywords:** Acamprosate, Hypersalivation

## Abstract

**Introduction:**

Mr. X, a 39-year-old man presented to us with a history of alcohol use from the last 12-15 years in a dependence pattern with tolerance, uncomplicated withdrawal symptoms and salience. He was detoxified, given parenteral thiamine supplements and oral lorazepam to reduce withdrawal symptoms. He was contemplating to quit alcohol and thus about 4-5 days after his detoxification, tablet acamprosate 1998 mg/day was added, in three divided dosages. He was discharged after 10 days and had no withdrawal signs or cerebellar deficits. In the next follow-up after two weeks, he reported to be abstinent from alcohol, but now complained of new onset coarse tremors and excessive salivation. He had no other extra pyramidal or cerebellar symptoms, no hepatic or renal dysfunction and no neurological deficits. The Patient had a drooling score of 6 on Drooling Severity and Frequency Scale(DSFS).

**Objectives:**

Acamprosate and naltrexone are the only two drugs approved by the US Food and Drug Administration for achieving abstinence in patients with Alcohol Dependence Syndrome. Acamprosate is well tolerated and has a few drug interactions. It has a comparatively benign side effect profile which includes diarrhea, intestinal cramps, itching, dizziness, muscle weakness, headache, flatulence, nausea, anxiety, and insomnia. Here we report hypersalivation and coarse tremors as unusual side effects of acamprosate.

**Methods:**

Cross-sectional

**Results:**

Here we report hypersalivation and coarse tremors as unusual side effects of acamprosate.

**Conclusions:**

The probable mechanism responsible for this is thought to be acamprosate induced decrease in dopamine release in ventral tegmental area due to diminished glutamate activity.

**Disclosure:**

No significant relationships.

